# Relative Biological Effectiveness of High LET Particles on the Reproductive System and Fetal Development

**DOI:** 10.3390/life10110298

**Published:** 2020-11-20

**Authors:** Bing Wang, Hiroshi Yasuda

**Affiliations:** 1 National Institute of Radiological Sciences, National Institutes for Quantum and Radiological Science and Technology, Chiba 263-8555, Japan; wang.bing@qst.go.jp; 2 Research Institute for Radiation Biology and Medicine, Hiroshima University, Hiroshima 734-8553, Japan

**Keywords:** space radiation, high LET, relative biological effectiveness (RBE), reproductive system, embryo, fetus, deterministic effect

## Abstract

During a space mission, astronauts are inevitably exposed to space radiation, mainly composed of the particles having high values of linear energy transfer (LET), such as protons, helium nuclei, and other heavier ions. Those high-LET particles could induce severer health damages than low-LET particles such as photons and electrons. While it is known that the biological effectiveness of a specified type of radiation depends on the distribution of dose in time, type of the cell, and the biological endpoint in respect, there are still large uncertainties regarding the effects of high-LET particles on the reproductive system, gamete, embryo, and fetal development because of the limitation of relevant data from epidemiological and experimental studies. To safely achieve the planned deep space missions to the moon and Mars that would involve young astronauts having reproductive functions, it is crucial to know exactly the relevant radiological effects, such as infertility of the parent and various diseases of the child, and then to conduct proper countermeasures. Thus, in this review, the authors present currently available information regarding the relative biological effectiveness (RBE) of high-LET particles on the deterministic effects related to the reproductive system and embryonic/fetal development for further discussions about the safety of being pregnant after or during a long-term interplanetary mission.

## 1. Introduction

Exposure to ionizing radiation (“radiation” hereafter) has been one of the major safety concerns in space missions [1,2,3,4,5,6]. Longer stay in the outer space environment will inevitably increase the health risks of astronauts due to the exposures to two major radiation sources: galactic cosmic rays (GCR) and solar particle events (SPEs). Both GCR and SPEs are mostly constituted from high-energy charged particles such as protons (85% on the fluence basis), helium nuclei (14%), and other heavier ions (1%) [7,8,9,10]. These particles produce various secondary radiations such as electrons, neutrons, recoiled nuclei and other smaller particles through the nuclear reactions in the walls of spacecraft, instruments and human bodies. In addition, large SPEs would significantly increase their exposures mainly to protons and their secondary radiations. Absorbed doses for the largest SPEs were estimated to be higher than 1 Gy [11] and could reach 10 Gy in a thinly-shielded spacecraft in deep space [12]. The present knowledge from radiobiology implies that this dose level of even low-LET particles could definitely cause significant biological effects. Thus, any astronauts involved in a future interplanetary exploration with a long duration up to three years would receive inevitably high radiological risks and they should be protected by proper countermeasures [13,14,15,16,17,18,19].

**Figure 1:**
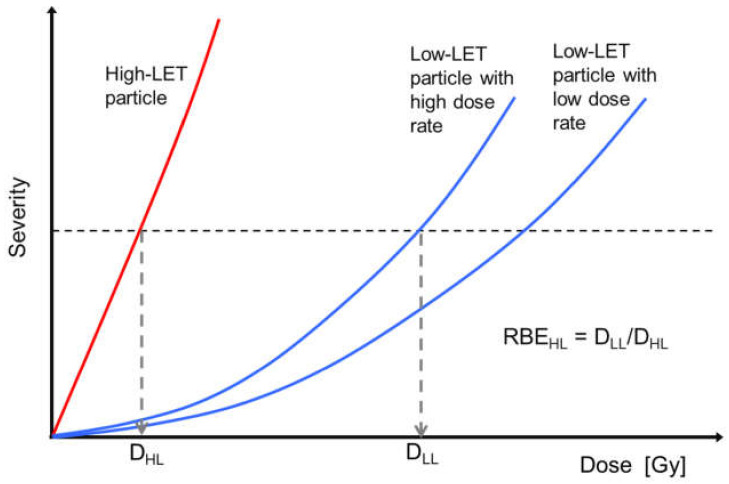
Diagram of the relationship between dose and severity of a deterministic effect. The RBE is calculated as the ratio of the high-LET particle dose (D_HL_) to the low-LET, reference particle dose (D_LL_) at the same level of severity for a specific biological endpoint. RBE values could vary depending on the particle species, dose rate/fractionation, severity level in respect, etc.

The radiation exposure of an astronaut is evaluated as the absorbed dose (D) weighted by quality factor (Q) or relative biological effectiveness (RBE) [2,6]; D is defined as the energy deposited per unit mass of the tissue; Q is a coefficient used for adjustment of radiation quality regarding stochastic effects (i.e., cancers and hereditary effects) and RBE is that regarding deterministic effects that are induced by relatively high doses. Both Q and RBE are given as functions of linear energy transfer (LET). The high-LET particles such as protons and heavy ions produce dense ionizations along the tracks and thus could induce larger biological effects than the low-LET particles such as X-rays and γ-rays [20,21,22]. In other words, those high-LET particles generally show RBE values higher than 1. A conceptual diagram of RBE-value determination is illustrated in Figure 1. As indicated there, RBE values could significantly change depending on combination of particle species, dose rate/fractionation, and selected level of severity/total dose, as well as the biological endpoint in respect. By lowering the targeted severity level, we could get a higher RBE values. Thus, it has been assumed that the Q values used for assessment of stochastic effects appearing after lower dose exposure would be higher than RBE values.

Since major components of both GCR and SPE have high LET values, it is desirable that the comprehensive knowledge base on the biological effects of the high-LET particles be established, so that the space agencies and astronauts could ensure that the associated health risks in any long-term space missions are low enough in view of radiological protection. As to low-Earth-orbit missions, the radiological risks have been long recognized and discussed based on two primary effects: late effects such as cancer and early effects such as the change of blood forming function with consideration of possible damages on the physiological function of central nervous system (CNS) [2,3,4]. However, the effects of space radiation exposure regarding reproductive functions have not been clearly identified. As it is probable that future interplanetary missions would involve some young astronauts who have reproductive abilities, it is desirable to collect scientifically sound information that are needed for reliable risk assessments on their possible pregnancies.

The tissue reactions of radiation exposure on the reproductive system are observed as general germ cell killing, chromosome damage in germ cells, germ cell morphological abnormalities, reproductive organ weight loss, etc. The resultant clinical symptoms include: infertility, embryonic and fetal death, stillbirths, genetic alteration, congenital malformations and other birth defects, growth retardation, mental retardation and other neurobiological effects, and cancer during pregnancy [23,24].

For low-LET particles, the detrimental effects other than cancer in the human embryo and fetus have been investigated in many experimental studies. The threshold doses for induction of those effects are briefly summarized as follows [23]: (1) in the period of the 1st to 2nd weeks postconception, 0.15–0.2 Gy for embryo lethality; (2) in the period of the 3rd to 5th weeks postconception, 0.25–0.5 Gy for embryo lethality, 0.2–0.5 Gy for permanent growth retardation in the adult and >0.5 Gy for gross anatomic malformation; (3) in the period of the 6th to 13th weeks postconception, >1 Gy for fetus lethality and 0.25–0.5 Gy for permanent growth retardation in the adult; (4) in the period of the 8th to 25th weeks postconception, >0.5 Gy for severe mental retardation and decrease in intelligence quotient (IQ) scores; (5) in the period of the 14th weeks postconception to term, >1.5 Gy fetus lethality and >0.5 Gy for gross anatomic malformation. On the other hand, there are still large uncertainties regarding the effects of exposure to high-LET particles on the embryo and fetus. It is presumed though that the threshold values of absorbed dose for the high-LET particles would be lower since they generally have the RBE values greater than the unity.

With this thought, the authors conducted a systematic literature review focusing on the RBE values of high-LET particles for the deterministic effects on the reproductive system and embryonic/fetal development. We targeted both epidemiological and experimental studies using both in vivo and in vitro systems. In the reviews of experimental investigations, the subjects were limited to the mammalians and mammals and, in collecting the data from in vitro studies, the subjects were limited to eggs and embryonic/fetal primary cultures except the established cell lines derived from embryos.

## 2. On the Reproductive System

The radiation-induced tissue reactions on the reproductive system such as germ cell killing and morphological abnormalities depend on the dose levels and developmental stages [23]. While convincing evidences of radiation-induced germline mutation manifesting as heritable disease are unavailable in humans, relevant translational effects due to exposure to radiations were demonstrated in some studies using mice.

### 2.1. Effects on Female Reproductive System

In humans, there are about 2 million germ cells in the ovary at birth. The number of follicles declines rapidly from about 4 hundred thousand at 12–16 years to 8 thousand at 40–44 years due to atresia. About 4 hundred oocytes are ovulated during a reproductive period of about 35 years. According to the reports of United Nations Scientific Committee on the Effects of Atomic Radiation (UNSCEAR) [25,26], human oocytes are radiosensitive while many oocytes can be lost without affecting fertility. A single dose up to 0.6 Gy of low-LET radiation does not induce significant deleterious effect on the reproductive function while a dose of 1.5 to 6 Gy could suppress ovulation. A dose from 3 to 8 Gy could cause permanent sterility. Radiosensitivity increased with age due to the decline in the oocyte pool size [27]. It was reported that the murine oocyte was more sensitive than the human [21].

#### RBE for Ovary

While it is known that the ovary is one of the most radiosensitive organs in regard to carcinogenesis [28], no RBE data on humans can be found. In mice, for killing of immature oocytes as the endpoint, the RBE value was obtained as 1.7 for 0.43 MeV neutrons [29], 1.6–3.5 for neutrons from ^252^Cf [30]. Exposure during the pre-implantation period and major organogenesis, the RBE value of fast neutrons with an energy of 0.4 and 14 MeV was 1.0 and 1.3, respectively [31].

The RBE value of β-rays from tritiated water (HTO) for the oocyte killing in mice was in the range of 1.1 to 3.5 [32]. When being exposed from conception to the first 2 weeks of lactation, the RBE value of the HTO β-rays was in the range of 1.6 to 2.8 [33,34].

The RBE value of ^239^Pu α-rays was about 2.5 for impairment of female fertility in mice [35].

For accelerated beams of neon (energy: 450 MeV/amu), argon (570 MeV/amu) and silicon (670 MeV/amu) ions, the RBE values were in the range of 0.4 to 0.6, 0.4 to 3.0 and 0.4 to 2.2, respectively, for early effects (cell depletion) on the ovary of mice [21]. The RBE value of accelerated carbon ions (80 MeV/amu, LET: 31 keV/µm) was in the range of 1.32 to 2.49 for induction of chromosomal aberrations in immature oocytes [36].

In Table 1, the RBE values of electrons (β-rays), neutrons, helium nuclei (incl. α-rays), carbon, neon, silicon, and argon ions for the ovary are summarized.

### 2.2. Effects on Male Reproductive System

Testis contains a wide range of cell types that are in different states of differentiation and with varied radiosensitivity. It is known that Type B spermatogonia is one of the most radiosensitive tissues, and 0.1 Gy X-rays could reduce the sperm count due to killing of the late-stage differentiating spermatogonia. An acute or fractionated dose up to 4 Gy could cause a temporary sterility. A dose higher than 6 Gy could cause a permanent sterility in men [26].

#### RBE for Testis

The RBE data for testis of humans are limited. In mice, for induction of sperm head abnormalities, the RBE values of Auger electron emitted by radionuclides (^125^I and ^111^In) were in the range of 2.5 ± 0.2 to 59 ± 4.0 [37]. For killing of spermatogonia, the RBE values of these Auger electron were in the range of 1.0 to 7.9 [37]. The RBE values of Auger and Coster-Kronig electrons from ^210^Tl relative to β-rays from ^201^Tl was 3.8 ± 0.4 for sperm head killing and testis weight loss as endpoints [38]. The RBE values of HTO β-rays ranged from 1.04 to 3.0 for induction of chromosome damage in human spermatozoa, depending on the absorbed dose [39].

The RBE value of α-rays emitted from ^210^Po was 245 ± 23 for induction of sperm head abnormalities and those for killing of spermatogonia was 6.7 [37]. The RBE value of 3.2–8.8 MeV α-rays was maximum of 7.4 for testicular sperm head survival as the biological endpoint [40,41,42].

In a study using ^252^Cf source, the RBE values of the fission neutrons for induction of chromosome damage including structural chromosome aberrations and chromosomal abnormality in human spermatozoa were reported as 1.6 to 3.9 [43]. For the induction of chromosome aberrations in spermatogonia and primary spermatocytes, the RBE value of fission neutrons (0.85 MeV) was in the range of 10 to 24 [44]. The RBE value of 0.4 MeV neutrons was 5.65 for induction of chromosome aberrations in secondary spermatocytes [45]. Generally, the RBE value of fission neutrons and neutrons with mean energy up to 50 MeV was in the range of 2.0 to 7.0 for induction of chromosome aberrations in spermatocytes [21,46,47]. The RBE value of ^252^Cf neutrons for the weight loss of mouse testis was 5.1 [48].

For the neutrons with energy of 1.0, 2.3, and 5.6 MeV, the RBE values were 5.7, 4.6, and 3.0, respectively, for depletion of spermatogonia [49]. The RBE values of neutrons with an energy of 5.5 MeV were 4.57 for depletion of spermatogonia and 4.25 for mouse testis weight loss [50].

The RBE value of accelerated helium ions (228 MeV/amu, LET: ~60 keV/µm) was 1.15–1.3 for cell killing of spermatogonia in mice [51]. The RBE values of carbon ions (50 MeV/amu) were 1.67 for induction of chromosomal aberrations in spermatogonia and 1.66 for spermatocytes [52]. The RBE values of accelerated oxygen ions (60 MeV/amu) were 1.84, 1.22 and 1.29, respectively, for testis weight loss, sperm count decrease and sperm abnormalities [53]. For accelerated carbon, neon and argon ions (400 to 670 MeV/amu), the RBE values of these high-LET particles for cell killing of spermatogonia showed a maximum of 3.0 and reduced when the LET exceeded about 100 keV/µm; for testis weight loss, the RBE values obtained were 2 for carbon ions, 2.2 for neon ions, and 3.0 for argon ions in mice [51].

Regarding exposure of fetal rats in the major organogenesis period to accelerated heavy charged particles, the RBE values of carbon ions (290 MeV/amu, LET: 13 keV/µm) and that of neon ions (400 MeV/amu, LET: 40 keV/µm) were in the range of 1.0 to 1.4 and 1.0 to 1.3, respectively, for induction of apoptosis in gonocytes; the RBE values of carbon ions and neon ions were 0.9 and 1.0, respectively, for reduction of breeding ability (the mean number offspring obtained by mating the prenatally irradiated males with the non-irradiated females) [54].

In Table 2, the RBE values of electrons (including β-rays), neutrons, helium nuclei (including α-rays), carbon, oxygen, neon, and argon ions for the testis are summarized.

## 3. On Embryonic and Fetal Development

Except for heritable diseases in certain animal models and cancers, the detrimental effects of radiation exposure include mainly embryonic death, fetal death, congenital malformations, microcephaly, growth retardation, mental retardation, decreased IQ, epilepsy, and neurobehavioral effects in humans. These deterministic effects have different threshold doses depending on the endpoints and the timing of exposure [23].

### 3.1. Effects on the Developing Embryo and Fetus

The developing embryo and fetus are extremely radiosensitive due to rapid cell proliferation, migration and differentiation. Response of each organ system depends on many factors related to both the radiation source and the physiological system, including radiation quality, quantity and dose rate, oxygen tension, cell type and the developmental stage at the time of exposure. Most of reliable data regarding the effects on human embryo and fetus have been obtained in high-dose exposure situations.

Congenital malformations occur during organogenesis; and the greatest probability in a specific organ system is with a critical period when the peak differentiation takes place. Malformations of central nervous system (CNS) are commonly divided into two groups, namely, the organogenetic and the histogenetic. The organogenetic malformations occur in the period of major organogenesis and the histogenetic malformations during the differentiation and growth of the brain mantle.

Radiological risks are related to the stage of development, being the most significant during organogenesis and the early fetal stage. The deterministic effect is with a threshold dose ≥100 mGy of low LET radiation, and most malformations at the 100 mGy threshold are CNS-related [28,55,56]. In brief, (1) in the period of pre-conception, there is no increased risk of malformations in children due to exposure of parental gonads; (2) in the pre-implantation stage (from fertilization to the 9th day), an all-or-none phenomenon shows either in utero death and resorption (usually undetected) or normal fetal risk, and the deterministic effect is with a threshold dose of about 100 mGy; (3) during the organogenesis stage (from the 3rd to 8th week after fertilization), there is a substantial decrease in fetal death but an increase in congenital malformations with the peak developmental periods of various organs and systems especially at 20–40 days, and an increase in growth retardation following exposure at >4 weeks after conception. There is little risk of mental retardation before the 8th week. The deterministic effect is with a threshold dose of >100 mGy; (4) In the fetal growth stage (from >8th week after fertilization until term), there is little risk of congenital malformations. A significant mental retardation risk is at the 8th to 25th weeks, with the highest risk (5) or more times greater than in later periods) in the period from the 8th to 15th week, corresponding to the time for the most rapid proliferation and migration of neurons to the neocortex. The frequency of severe mental retardation is approximately proportional to the absorbed dose, and an increased risk of growth retardation occurs after 25th weeks. There is an apparent dose-related reduction in mean IQ in the period from the 8th to 15th week and the 16th to 25th week.

#### RBE for Embryonic and Fetal Development

No adequate human data on utero exposure are available to define the RBE values of high-LET particles (incl. neutrons) for induction of malformations, mental deficits, cataract and cardiovascular diseases.

Exposure of utero to β-rays from tritium during the whole period of pregnancy, the RBE value was of 1–2 using chromosomal aberrations in bone marrow cells in postnatal mice as the endpoint [57].

For embryo survival, the RBE value of neutrons with energy ranging from 1 to 800 MeV was 48 in Japanese medaka fish [58]. The RBE value of 150 MeV protons was reported to be 1.1 to 1.2 in zebrafish [59]. In mice during the pre-implantation period at the early zygote stage, the RBE value of cyclotron neutrons with a mean energy about 7 MeV was 2.3 for prenatal mortality, and 2.0 to 2.8 for malformed fetuses [60]. The RBE value of neutrons with an energy of 5.8 to 6.0 MeV was 2.0 to 3.7 for malformed fetuses [61].

In cultured pre-implantation mouse embryos, using embryo survival as the endpoint, the RBE value of neutrons was in the range of 2.0 to 10 [31,62,63], and the RBE value of β-rays from HTO was in the range of 1.0 to 1.7 [64]. Using chromosome aberrations, the RBE values of β-rays from HTO were 1.6 to 2.0 [65]. The RBE values of 6.0 MeV neutrons were 4.7, 4.8 and 7.4, respectively, for chromosomal anomalies at the first, second, and third mitosis after irradiations in one-cell mouse embryos [66].

In cultured fetal mouse midbrain cells and using varied endpoints such as cell proliferation, differentiation, cellular DNA and protein contents, the RBE value of β-rays from organically bound tritium compounds (i.e., methyl-^3^H-thymidine) was in the range of 4.6 to 8.7 [67]. The RBE values of cyclotron neutrons (7.0 MeV) for micronucleus induction in pre-implantation embryos were in the range of 2.5 to 3.5 [68]. For pre- and post-natal developmental defects including formation of micronuclei, lethality, malformations, weight defects, brain structure changes in experimental studies in mice, the RBE value of fast neutrons summarized by ICRP [31] was in the range of 1.8 to 7.4 with the mean value of 3.65. In rats, the RBE value of neutrons (0.43 MeV) was within this range with qualitatively and quantitatively different effects at least in parts [69]. The RBE value of neutrons (6 MeV) for brain anatomical defects in mice was with an average of 3.0 [31,70].

For exposure in organogenesis period, the RBE value of iron particles (500 MeV/amu, LET: 200 keV/µm) was in the range of 3.7 to 4.2. for induction of apoptosis in the developing optic tectum in medaka fish [71]; the RBE value of neutron (peak energy: 10 MeV) was a maximum of 9.8 for induction of neuron apoptosis in cerebral cortex in fetal mice [72]. Using prenatal development and postnatal neurophysiological accomplishment as the endpoints, the RBE value of β-rays from HTO ranged from 2.3 to 3.0 in mice and rats [54,73]. Using organ malformation as the endpoint, the RBE value of neutrons from ^252^Cf was in the range of 2.3 to 3.1 [30,74]. The RBE value of helium (530 MeV/amu, 4–6% LET: >40 keV/µm) was in the range of 1.0 to 1.4 for fetal killing effect in rats after exposure in early organogenesis period, showing higher values under hypoxic condition [75]. Exposure of fetal rats during major organogenesis period to accelerated heavy particles resulted in detrimental effects on prenatal development and postnatal neurophysiological accomplishment, the RBE value of carbon ions (290 MeV/amu, LET: 14 keV/µm) and neon ions (400 MeV/amu, LET: 40 keV/µm) was in the range of 1.0 to 2.04 and that of 1.0 to 2.14, respectively (here, the RBE values were estimated based on the data of Wang et al. [76,77,78]).

In Table 3, the RBE values of electron (β-rays), neutrons, protons, helium nuclei, carbon, neon and iron ions for embryonic and fetal developments are summarized.

### 3.2. Effects on Developing Brain

The developing brain is more susceptible to radiation than most other embryonic and fetal structures due to its architectural complexity and long developmental period, the vulnerability of the undifferentiated neural cells, the dependence of neuronal function on the position and migration of the neuronal cell, and the inability to replace lost neurons. Radiation exposure could induce mitotic death of glial or/and neuronal precursors, or kill post-mitotic but still immature neurons; intrude on migration via altering cell surface properties or killing the glial cells that guide the migrating neurons; impair capacity of the neurons to connect correctly; and accelerate or alter programmed cell death, essential to the normal development of brain and its adnexa.

Studies on survivors exposed in utero to atomic bombing of Hiroshima and Nagasaki focused on severe mental retardation, small head size, and reduced IQ scores. Human epidemiological studies supported a threshold of ≥300 mGy for severe mental retardation after exposure in the period from the 8th to 15th week [28].

### 3.3. Effects on Adult-Onset Noncancer Diseases

Long-term effects of prenatal radiation exposure are important to assess radiation risk [79]. In humans, epidemiological investigations including case report study showed induction of (cortical) cataract by irradiation of human fetus with doses used in medical therapeutics between 4 to 11 weeks and 30 to 33 weeks of gestation, and higher rates of congenital cataracts and truncus arteriosus in Marshallese infants [80,81,82]. For experimental studies in mouse models, X-ray irradiation with about 0.09 Gy of pre-cleavage ovum appeared statistically cataractogenic [83,84].

Among the atomic bomb survivors exposed in utero, positive effect on systolic hypertension in adolescence was reported [85]; cardiovascular disease risk was of great concern and cohort follow-up studies continued [86]. On the other hand, exposure in the organogenesis period showed heart proteome alterations and cardiac impairment in mice [87].

## 4. On Other Effects Related to Reproductive Ability

### 4.1. Lowering Fecundability

Though it has been reported that United States female astronauts have poor fecundability [88,89], it is hard to associate this observation with their exposures to space radiation. So far, we cannot find any data that assured the adverse effects of high-LET particles (i.e., main components of space radiation) on the reproductive abilities of the female astronauts. The observed lowering fecundability rather attributes to the older age of pregnancy [88], the occupational stress that could be significant during a space mission [89,90] and also to the small number of female astronauts who got pregnant after experiencing space flights [88].

While the contribution of space radiation to the observed low fecundability is unclear, together with other potentially hazardous factors, such as microgravity, hypergravity, circadian rhythm disruption, dehydration, limited food, altered environmental biota, and psychological stresses, exposure to space radiation could be a critical stressor affecting the reproductive health of astronauts [91,92,93]. We thus hope that the RBE data presented here will be useful for facilitating relevant studies to obtain evidence of the radiological effects of high-LET particles on the reproductive abilities that must be of more serious concern in future longer-term interplanetary missions than in the LEO missions.

### 4.2. Sexual Dysfunction

Exposure to radiation could induce sexual dysfunction (SD) in humans. Recent epidemiological studies have indicated a high prevalence of SD among the genitourinary cancer patients treated with radiotherapy, no matter their genders [94,95,96,97,98]. These studies have reported various SD symptoms such as decreased sexual desire, decreased intercourse frequency, erectile dysfunction (ED), premature ejaculation, inability to achieve orgasm, dyspareunia, vaginismus, and low satisfaction. The degrees of those symptoms are dependent on personal factors such as age, pretreatment potency, sexual orientation and medical treatment.

For men, erectile, ejaculatory and orgasm dysfunction are common side effects associated with prostate radiotherapy [99,100,101,102,103]. It is considered that morphologic arterial damage with aberrant alterations in internal pudendal arterial tone and reduction of motor function in the cavernous nerve due to axonal degeneration may contribute to ED. A reduction of testosterone level after irradiation may also affect the desire for sexual activity.

For women, exposure of the pelvic area to therapeutic radiations could affect their sex life due to inflammatory reactions of vagina and damage to ovary after the treatment (i.e., vaginal ulcers and radiation-induced menopausal symptom) [104,105,106]. Meanwhile, the etiology of the SD is often multifactorial and complicated, resulting from combinations of neurogenic, hormonal, muscular, as well as psychogenic causes.

In both men and women, damages to some organs such as bladder, colon and rectum could also impact their sexual functions.

The radiation-induced SD in humans is unlikely to be induced by a relatively low dose (~few Gy) that astronauts normally experience in any missions including the planned interplanetary missions. For example, the threshold dose for avoiding ED was probable to be in the range from 40 to 50 Gy [107,108,109,110]; this level is much higher than the highest absorbed dose (~ 10 Gy) predicted even for a long-term deep space mission. It should be noted, however, that we have no epidemiological or experimental data on various SD symptoms induced by high-energy, high-charge particles and neutrons that are common in space. It is desirable to collect more relevant data to define the RBE values of those high-LET particles that generate locally highly dense ionizations.

## 5. Summary

In this review, the authors presented the RBE values of some high-LET particles that astronauts will encounter in space for the reproductive system (see Table 1 and Table 2) and those for embryonic and fetal developments (see Table 3). The levels of radiation doses employed in the reviewed studies were considered to be adequate in light of the estimated doses (~10 Gy) that astronauts could receive from the largest SPEs in deep space [11,12].

Through the comprehensive literature survey, we found that the RBE values of high-LET particles vary widely, depending on various factors such as characteristics of the radiation source (particle species, dose and dose rate/fractionation), targeted biological endpoint and the severity in respect. In general, the results of in-vitro studies using cultured cells indicated that: (1) increasing LET differentially reduced the shoulder region of the survival curve; (2) the single-irradiation RBE values of high-LET particles increased with decreasing dose; (3) the RBE value was less than that calculated from two isoeffective (total) responses for multiple fractionated doses or at low dose rates; and (4) the RBE values increased with increasing LET of incident particles up to 100 keV/µm. The decrease of RBE value for higher LET region is attributable to the so-called “overkill effect” which means the lowering efficiency of a unit-dose energy deposition for induction of a certain biological effect [31]; the highly-dense energy deposition from a high-LET particle along the track is not so efficient in producing DNA stand breaks or other microscopic biological damages.

The authors found that many of the RBE values were collected from laboratory studies using small animals such as mice and rats due to the limitation of human data from clinical studies on patients or epidemiological studies on atomic bomb survivors. It is thus important to manage carefully the species-dependent differences when applying those findings to the ongoing radiological risk assessments for astronauts. Nevertheless, we believe that those data obtained by using animals should provide some, at least qualitative, relevant information for humans as long as appropriate experimental procedures were followed at comparable stages in the respective organ development. It is expected that further spread of hadron therapy treating genitourinary cancers will provide both useful experimental data and convincing theoretical methods to determine accurate RBE values for humans. In any ways, it is critically important to design and perform carefully descriptive and comparative animal-model studies with intention of applying the obtaining data to humans.

The authors hope that this review will be useful for the evaluation and reduction of pregnancy-related risks of the astronauts who have reproductive functions and plan to be involved in future interplanetary missions, expecting that further efforts will be actively made to collect relevant information regarding the combined effects of high-LET space radiation and gravity changes, e.g., [111,112,113,114,115,116], and theoretical based on the differences of the RBE values between low-LET and high-LET particles, e.g., [117], and also those of between low and high dose rates, e.g., [118].

Reproducibility is a substantial nature for all living organisms including human beings. Sex, pregnancy, reproduction, and the expansion of families have been attractive themes in scientific research. Furthermore, with the ongoing climate changes and spreading infectious diseases, science for saving humankind and their civilization from extinction will be more important in next decades. Investigations for achieving a perfectly safe interplanetary mission would facilitate revolutions in the relevant sciences. Actually, it has been indicated that colonizing other parts of the solar system should prevent the humankind from being exterminated by extinction events [119]. Along this direction, radiobiology in space, including the challenge to maintain the reproducibility of humans in deep space, will be crucial for our future as well as the success of a manned mission to Mars.

## Figures and Tables

**Table 1:** The RBE values of selected incident particles for ovary.

Particle	Source or Energy (LET ^1^)	Biological Endpoint	RBE Value		
			of the Particle for the Specific Endpoint	of the Same Particle for All Endpoints	of All the Particles for All Endpoints
Electron	β-rays from HTO	Oocyte killing in mice	1.1–3.5 [32]	1.1–3.5	0.4–3.5
	β-rays from HTO	Oocyte killing in mice	1.6–2.8 [33,34]		
Neutron	0.4 MeV	Oocyte killing in mice	1.0 [31]	1.0–3.5	
	0.43 MeV	Oocyte killing in mice	1.7 [29]		
	14 MeV	Oocyte killing in mice	1.3 [31]		
	from ^252^Cf	Oocyte killing in mice	1.6–3.5 [30]		
Helium	α-rays from ^239^Pu	Impairment of fertility in mice	2.5 [35]	2.5	
Carbon	80 MeV/amu (31 keV/µm)	Oocyte killing in mice	1.32–1.49 [36]	1.3–1.5	
Neon	450 MeV/amu (>30 keV/µm)	Oocyte killing in mice	0.4–0.6 [21]	0.4–0.6	
Silicon	670 MeV/amu (>50 keV/µm)	Oocyte killing in mice	0.4–3.0 [21]	0.4–3.0	
Argon	570 MeV/amu (>85 keV/µm)	Oocyte killing in mice	0.4–2.2 [21]	0.4–2.2	

^1^ Some of the LET values were estimated from the incident beam energies.

**Table 2:** The RBE values of selected incident particles for testis.

Particle	Source or Energy (LET ^1^)	Biological Endpoint	RBE Value		
			of the Particle for the Specific Endpoint	of the Same Particle for All ENDPOINTS	of All the Particles for All Endpoints
Electron	Auger electrons from ^125^I and ^111^In	Sperm head abnormalities in mice	2.3–63 [37]	1.0–63	0.9–270
	Auger electrons from ^125^I and ^111^In	Spermatogonium killing in mice	1.0–7.9 [37]		
	β-rays from HTO	Spermatozoa chromosome damage in humans	1.04–3.0 [39]		
Neutron	from ^252^Cf	Spermatozoa chromosome damage in humans	1.6–3.9 [43]	1.6–24	
	from ^252^Cf	Testis weight loss in mice	5.1 [48]		
	0.4 MeV	Chromosome aberrations in secondary spermatocytes in mice	5.65 [45]		
	0.85 MeV	Chromosome aberrations in spermatogonia and primary spermatocytes in mice	10–24 [44]		
	1 MeV	Spermatogonium killing in mice	5.7 [49]		
	2.3 MeV	Spermatogonium killing in mice	4.6 [49]		
	5.5 MeV	Testis weight loss in mice	4.25 [50]		
	5.5 MeV	Spermatogonium killing in mice	4.57 [50]		
	5.6 MeV	Spermatogonium killing in mice	3 [49]		
	~50 MeV	Chromosome aberrations in spermatocytes in mice	2.0–7.0 [21,46,47]		
Helium	α-rays (3.2–8.8 MeV)	Sperm head killing in mice	~7.4 [40,41,42]	~270	
	α-rays from ^210^Po	Sperm head abnormalities in mice	245 ± 23 [37]		
	α-rays from ^210^Po	Spermatogonium killing in mice	6.7 [37]		
	228 MeV/amu (~60 keV/µm)	Spermatogonium killing in mice	1.15–1.3 [51]		
Carbon	50 MeV/amu (>45 keV/µm)	Chromosomal aberrations in spermatogonia in mice	1.67 [52]	0.9–3.0	
	50 MeV/amu (>45 keV/µm)	Chromosomal aberrations in spermatocytes in mice	1.66 [52]		
	400 to 670 MeV/amu (>11 keV/µm)	Spermatogonium killing in mice	<3 [51]		
	400 to 670 MeV/amu (>11 keV/µm)	Testis weight loss in mice	2 [51]		
	290 MeV/amu (13 keV/µm)	Gonocyte killing in mice	1.0–1.4 [54]		
	290 MeV/amu (13 keV/µm)	Impairment of fertility in mice	0.9 [54]		
Oxygen	60 MeV/amu (>70 keV/μm)	Testis weight loss in mice	1.84 [53]	1.2–1.8	
	60 MeV/amu (>70 keV/μm)	Sperm count decrease in mice	1.22 [53]		
	60 MeV/amu (>70 keV/μm)	Sperm abnormalities in mice	1.29 [53]		
Neon	400 to 670 MeV/amu (> 30 keV/µm)	Spermatogonium killing in mice	<3 [51]	1.0–3.0	
	400 to 670 MeV/amu (>30 keV/µm)	Testis weight loss in mice	2.2 [51]		
	400 MeV/amu (40 keV/µm)	Gonocyte killing in mice	1.0–1.3 [54]		
	400 MeV/amu (40 keV/µm)	Impairment of fertility in mice	1 [54]		
Argon	400 to 670 MeV/amu (>80 keV/µm)	Spermatogonium killing in mice	~3 [51]	~3.0	
	400 to 670 MeV/amu (>80 keV/µm)	Testis weight loss in mice	3 [51]		

^1^ Some of the LET values were estimated from the incident beam energies.

**Table 3:** The RBE values of selected incident particles for embryonic and fetal development.

Particle	Source or Energy (LET ^1^)	Biological Endpoint	RBE Value		
			of the Particle for the Specific Endpoint	of the Same Particle for All Endpoints	of All the Particles for All Endpoints
Electron	β-rays from HTO	Chromosomal aberrations in bone marrow cells in mice	1–2 [57]	1.0–8.7	1.0–48
	β-rays from HTO	Embryo killing in mice	1.0–1.7 [64]		
	β-rays from HTO	Chromosome aberrations in embryo cells in mice	1.6–2.0 [65]		
	β-rays from HTO	Cell proliferation, differentiation, cellular DNA and protein contents in fetal midbrain in mice	4.6–8.7 [67]		
	β-rays from HTO	Impairment of prenatal development and postnatal neurophysiological accomplishment in mice and rats	2.3–3.0 [54,73]		
Neutron	from ^252^Cf	Malformation in mice	2.3–3.1 [30,74]	2.3–48	
	0.43 MeV	Formation of micronuclei, lethality, malformations, weight defects, brain structure changes in mice,	1.8–7.4, 3.65 in average [69]		
	1–800 MeV	Embryo killing in medaka fish	48.1 [58]		
	7 MeV	Prenatal mortality in mice	2.3 [60]		
	7 MeV	Malformation in mice	2.0–2.8 [60]		
	5.8–6.0 MeV	Malformation in mice	2.0–3.67 [61]		
		Embryo killing in mice	2.0–10 [31,62,63]		
	6.0 MeV	Chromosomal anomalies at the first mitosis in one-cell embryos in mice	4.7 [66]		
	6.0 MeV	Chromosomal anomalies at the second mitosis in one-cell embryos in mice	4.8 [66]		
	6.0 MeV	Chromosomal anomalies at the third mitosis in one-cell embryos in mice	7.4 [66]		
	6.0 MeV	Brain anatomical defects in mice	3 in average [31,70]		
	7.0 MeV	Micronucleus induction in pre-implantation embryos in mice	2.5–3.5 [68]		
	Peak energy: 10 MeV	Induction of neuron apoptosis in fetal cerebral cortex in mice	9.8 [72]		
Proton	150 MeV	Embryo killing in zebrafish	1.13–1.2 [59]	1.1–1.2	
Helium	530 MeV/amu (4–6% >40 keV/µm)	Fetal lethality in mice	1.0–1.4 [75]	1.0–1.4	
Carbon	290 MeV/amu (14 keV/µm)	Impairment of prenatal development and postnatal neurophysiological accomplishment in rats	1.0–2.04 [76,77,78]	1.0–2.0	
Neon	400 MeV/amu (40 keV/µm)	Impairment of prenatal development and postnatal neurophysiological accomplishment in rats	1.0–2.14 [76,77,78]	1.0–2.1	
Iron	500 MeV/amu (200 keV/µm)	Induction of apoptosis in the developing optic tectum in medaka fish	3.7–4.2 [71]	3.7–4.2	

^1^ Some of the LET values were estimated from the incident beam energies.
